# Family Income Reduces Risk of Obesity for White but Not Black Children

**DOI:** 10.3390/children5060073

**Published:** 2018-06-10

**Authors:** Shervin Assari

**Affiliations:** 1 Department of Psychology, University of California, Los Angeles (UCLA), Los Angeles, CA 90095, USA; assarish@ucla.edu; Tel.: +1-734-232-0445; 2 BRITE Center, University of California, Los Angeles (UCLA), Los Angeles, CA 90095, USA; 3 Center for Research on Ethnicity, Culture and Health, School of Public Health, University of Michigan, Ann Arbor, MI 48109, USA; 4 Department of Psychiatry, University of Michigan, Ann Arbor, MI 48109, USA

**Keywords:** obesity, body mass index, socioeconomic status, income, ethnic groups, Blacks, ethnicity

## Abstract

*Background:* Although the protective effects of socioeconomic status (SES) on obesity and cardiovascular disease are well established, these effects may differ across racial and ethnic groups. *Aims:* Using a national sample, this study investigated racial variation in the association between family income and childhood obesity in White and Black families. *Methods:* This cross-sectional study used data from the National Survey of Children’s Health (NSCH), 2003–2004, a nationally representative survey in the United States. This analysis included 76,705 children 2–17 years old who were either White (*n* = 67,610, 88.14%) or Black (*n* = 9095, 11.86%). Family income to needs ratio was the independent variable. Childhood obesity was the outcome. Race was the focal moderator. Logistic regression was used for data analysis. *Results:* Overall, higher income to needs ratio was protective against childhood obesity. Race, however, interacted with income to needs ratio on odds of childhood obesity, indicating smaller effects for Black compared to White families. Race stratified logistic regressions showed an association between family income and childhood obesity for White but not Black families. *Conclusions:* The protective effect of income against childhood obesity is smaller for Blacks than Whites. Merely equalizing population access to SES and economic resources would not be sufficient for elimination of racial disparities in obesity and related cardiovascular disease in the United States. Policies should go beyond access to SES and address structural barriers in the lives of Blacks which result in a diminished health return of very same SES resources for them. As the likely causes are multi-level barriers, multi-level interventions are needed to eliminate racial disparities in childhood obesity.

## 1. Introduction

Despite the well-established effects of socioeconomic status (SES) on health [1,2,3,4,5,6,7,8,9,10], health gains that follow access to SES resources vary across racial and demographic groups [11,12]. Household income, parental education, and parental employment are protective of health problems among children [13,14], while poverty and financial strain are major risk factors for health problems among children [15]. Childhood obesity is a major consequence of poverty [16].

The protective effects of SES on population health [8,9,10], however, vary across sub-populations [11,12,17]. Blacks and Whites differ in the effect of SES on obesity [17]. This is because populations differ in their readiness to translate their resources to tangible outcomes [18,19]. Although, overall, high SES reduces population exposure to risks [8,9,10], this impact is systemically smaller for minority than majority groups [20,21,22]. As a result, the very same SES indicators such as education and employment may have smaller effects on purchasing power in racial minority groups [23,24,25,26]. How high SES increases a population’s access to resources and improves health seeking behaviors [8,9,10,27] also depends on race [28]. Due to differential treatment by society, the very same SES generates smaller changes in access to material and human resources for Blacks than Whites [29,30]. Upward social mobility may also be associated with extra psychological and physiological costs for minority populations [18,21,22,23,31,32,33,34,35]. To climb the social ladder, Blacks may turn to effortful coping for upward social mobility that increases risk of psychological burn out [36,37]. Due to scarcity of educational resources in urban areas, public education is of lower quality for Blacks, which, in turn, results in smaller protective effects of education on the lives of Blacks compared to Whites [28,38]. This phenomenon, defined as smaller health effects of SES indicators across minority groups [17,39,40,41] is also called Minorities’ Diminished Return theory [11,12].

Minorities’ Diminished Return theory has received considerable empirical support [11,12]. Protective effects of education on drinking patterns [42], depressive symptoms [43], suicidality [44], chronic disease [43], and mortality [45,46,47,48] are shown to be smaller for Blacks than for Whites. In some instances, not only does high SES not protect Blacks, but it may even increase the risk of poor mental health for Blacks. For instance, income is positively associated with risk of Major Depressive Disorder (MDD) for Black boys [49] and men [35,50]. High education may increase the risk of suicide for Black women [44] and future risk of depressive symptoms for Black men [43]. This might be in part because high SES Black families experience more discrimination than low SES Black families [18].

Racial disparity in obesity is one of the main contributors to racial disparities in cardio-metabolic disease as well as mortality [51]. Three main mechanisms have explained racial disparities in obesity: SES differences [52], behavioral differences [52], and environmental differences [53]. Obesity increases the risk of a wide range of undesired health outcomes such as heart disease, stroke, hypertension, and diabetes [54]. Black–White differences in obesity may partially explain some of the racial disparity in cardiovascular and all-cause mortality between Blacks and Whites [55]. This is partly because obesity during childhood as well as adulthood is a major gateway to several non-communicable diseases that are disproportionately more common in Blacks [56,57]. While mediating effects of upstream determinants such as SES and behaviors such as exercise have received attention as a mechanism for obesity disparities [52], less is known about the differential effects of social determinants.

In two studies [58,59] data from Exploring Health Disparities in Integrated Communities-SWB (EHDIC-SWB) and National Health Interview Survey were compared for disparities in obesity between White and Black men and women. In the national, but not EHDIC-SWBl sample, Black women [58] and Black men [59] were at a higher risk of obesity than their White counterparts. These findings suggest that race disparities are limited to higher levels of SES, while there are no race disparities in obesity among low SES people who share the same social context [58,59]. These studies suggest that race may interact with SES on risk of obesity.

To better understand the sub-population heterogeneity in the protective effect of SES on childhood obesity, this study used a national sample to examine racial differences in the effects of family income on childhood obesity in the United States.

## 2. Materials and Methods

### 2.1. Design and Setting

With a cross-sectional design, this investigation used data from the National Survey of Children’s Health (NSCH), a study sponsored by the Maternal and Child Health Bureau and the National Center for Health Statistics. NSCH was one of the state-of-the-art studies to produce national and state-level prevalence estimates of a variety of physical, emotional, and behavioral indicators of children’s health. The study also collected information on child’s family context and neighborhood [60,61,62].

### 2.2. Sampling and Participants

Similar to other national studies such as the National Immunization Study [60,61,62], NSCH used the State and Local Area Integrated Telephone Survey (SLAITS) program for the sampling frame [60,61,62]. To briefly describe the study sampling procedure, trained interviewers called telephone numbers at random to identify households with at least one child under the age of 18. From eligible households with one or more child, one child was randomly selected for the interview. The study also included an interview with the adult in the household who knew the most about the child’s health and well-being. After exclusion due to ineligible age, missing body mass index (BMI), income to needs ratio, and race/ethnicity, our analytic sample consisted of 76,705 children 2–17 years old (Whites, *n* = 67,610, Blacks, *n* = 9095).

### 2.3. Interviews

The study conducted an overall number of 102,353 interviews. All the interviews were completed between January 2003 and July 2004 and were performed either in English or Spanish. Trained interviewers asked parents or guardian respondents a series of questions regarding the physical, emotional, and behavioral health of their child, access to health care, parental health, and neighborhood characteristics [60,61,62].

### 2.4. Variables

The study evaluated the following variables: race, socioeconomic status (SES), gender, age, and obesity status.

#### 2.4.1. Race

To ensure the confidentiality of the participants, the NSCH collapsed responses to the question about the child’s race into White only, African American or Black only, other races, and multiple races. In the current study, we only used Blacks and Whites. We did not include the other race, and multiple race categories. Data were not available on the Hispanic Latino ethnicity.

#### 2.4.2. Income to Needs Ratio

Interviewers asked parents/guardians about household income. Income to household size was based on the Department of Health and Human Services federal poverty guidelines [60,61,62,63]. Although it was measured as an 8 level ordinal variable (less than 100% federal poverty level, 100% to below 133% federal poverty level, 133% to below 150% federal poverty level, 150% to below 185% federal poverty level, 185% to below 200% federal poverty level, 200% to below 300% federal poverty level, 300% to below 400% federal poverty level, and at or above 400% federal poverty level), SES was operationalized as a continuous measure ranging from 1 to 8, with higher score indicating higher SES [60,61,62,63].

#### 2.4.3. Parental Education

Interviewers asked parents/guardians about the highest educational attainment of the parents. Responses included: (1) less than high school graduate; (2) high school graduate; and (3) more than high school. The measure ranged from 1 to 3, with a higher score indicating higher educational attainment. 

#### 2.4.4. Childhood Obesity

Obesity status was a dichotomous variable calculated based on BMI which itself was calculated from parents or guardians reports on height and weight of the child. Parents and the guardians were asked the following two questions: “How tall is your child now?” and “How much does your child weigh now?”; BMI based on self-report strongly correlates with BMI based on measurements [64,65,66,67,68]. BMI was calculated as weight in kilograms divided by height in meters squared. Obesity status was defined according to the Centers for Disease Control and Prevention (CDC), which created gender- and age-specific growth charts that are used for children and adolescents aged 2 to 20 years [60,61,62,63]. A child with a BMI in the ≥95th percentile is considered obese [69]. We reclassified the pre-coded variable (four BMI classes) in the NSCH data set to a two level variable (obese vs. non-obese), according to the Centers for Disease Control and Prevention (CDC) growth charts.

### 2.5. Ethics

The NSCH protocol was approved by the CDC’s Institutional Review Board. All adolescents’ parents or legal guardian provided informed consent. Assent was obtained from adolescents. More information on ethical aspects of the study is available in [70]. All procedures performed in NSCH were in accordance with the ethical standards of the institutional and national research committee and with the 1964 Helsinki declaration and its later amendments or comparable ethical standards. Parental consent was obtained from parents. Assent was received from all participating children. The current study was exempt from Institutional Review Board review as the study was based on an anonymous public use data set with no identifiable information on the survey participants.

### 2.6. Data Analysis

Sampling Weights: The public-use NSCH data set provided the sampling weights used in these analyses. These weights consist of a base sampling weight and adjustment for multiple telephone lines per household and for non-response [60,61,62,63]. The weights are post-stratified so that the sum of weights for each state equals the number of children in that state as estimated by the July 2003 US census [60,61,62,63].

To account for the complex survey design of the NSCH data, we used Stata 13.0 (Stata Corp., College Station, TX, USA). We applied the Taylor series approximation technique for the estimation of complex design-based standard errors (SE) and variance. Percentages reported in this study are weighted thus they reflect nationally representative estimates.

For descriptive purposes, we reported frequency tables (%) and mean (SD). For bivariate analysis, we used Pearson correlation test in the pooled sample and in each race. We ran multiple regression models, first in the pooled sample and then in each race. In the pooled sample, first we ran models that only included main effect of SES and race. Then we ran a model that also included the race × SES interaction term. In all models, childhood obesity was the dependent variable, families’ income to needs ratio was the independent variable, and age, gender and education were covariates. Adjusted unstandardized b (regression coefficients) and their 95% confidence interval (CI) were reported. *p* values less than 0.05 were considered statistically significant.

## 3. Results

### 3.1. Descriptive Statistics

This analysis included 76,705 children 2–17 years old. The sample was composed of White children (*n* = 67,610, 88.14%) and Black children (*n* = 9095, 11.86%).

Table 1 summarizes the descriptive statistics for the pooled sample, as well as White and Black children. As this table shows, Black children were from families with lower education, lower income, and were at higher risk of childhood obesity.

### 3.2. Bivariate Associations

Table 2 summarizes the bivariate associations in the pooled sample as well as for Whites and Blacks. As this table shows, family income to need ratio was negatively associated with childhood obesity status in the pooled sample. This association was also significant for White but not Black children. 

### 3.3. Pooled Sample Logistic Regressions

Table 3 shows the results of two logistic regressions, one without interactions, and one with race by SES interactions. *Model 1* showed that, in the pooled sample, higher income to needs ratio was associated with lower odds of childhood obesity. *Model 2* showed an interaction between the effects of race and income to needs ratio on odds of childhood obesity, suggesting that the protective effect of income to needs ratio on odds of childhood obesity is smaller for Blacks compared to Whites. 

### 3.4. Race Stratified Logistic Regressions

Table 3 also shows the results of two logistic regressions specific to race. *Model 3* showed an association between income to needs ratio and childhood obesity for White children. *Model 4*, however, did not show any association between income to needs ratio and childhood obesity for Black children.

## 4. Discussion

We found Black–White differences in the effects of family income on childhood obesity. Black children’s risk of obesity was not dependent on family income. This pattern was totally different from White children in whom income had a protective effect against obesity.

As this study shows, the very same SES indicator is protective against childhood obesity for the socially privileged (White) group but not the economically disadvantaged (Black) group. This finding extends recent literature from adults [43,44] and older adults [17,28] to children. Although the exact mechanism for these differential gains are not clear, they seem to start early in life and be responsible for some of the health disparities in childhood [50,71]. That is, differential health effects of family SES is an early mechanism behind the indirect effect of race on health of the offspring.

Our findings successfully replicated the results of a recent study [71]. That study focused on the Black–White differences in the protective effect of family SES and family structure at birth on subsequent BMI at age 15 that followed 1781 youth from birth to age 15. The study revealed race by family SES and race by family structure interactions on BMI, indicating smaller effects for Blacks compared to Whites. Race by gender stratified regressions also showed more consistent patterns of associations between family SES and future BMI for Whites than Blacks. The study was one of the first to show that Minorities’ Diminished Return theory also holds for youth BMI [71]. In another study by Fradkin et al., in the pooled sample, youth in the highest SES had low prevalence of obesity than those of lower SES. This association could be confirmed for Hispanic and White youth, but not for Black youth [72].

We should declare that the findings do not suggest that Blacks are unable to efficiently use their available SES resources and turn them to tangible health outcomes, as that would be *victim blaming* [73]. Instead, it is the U.S. social structure, segregation, and structural racism to be blamed for these diminished returns [11,12]. The disproportionately high rate of societal and structural barriers in the lives of Blacks and other minority groups hinder Black families’ ability to gain health from their SES resources. In other words, it is the American social system that fails by charging Black families extra cost to climb the social ladder. Upward social mobility is more challenging for the minority people [21,22]. American society is constantly maximizing the gain of the privileged group and minimizing those of minorities [11,12]. This may be why U.S. is experiencing an increase in the Black–White economic gap.

Despite hard work and high aspirations, high SES Black families continue to face systematic barriers that increase their stress and reduces their chance of developing positive health outcomes. High SES Black families report higher levels of discrimination that reduce the expected health gains [43]. We argue that, in a race aware society, and in the presence of racism and discrimination (an environment which is difficult to control), high aspirations may even be detrimental to the health of Blacks [18,49]. Hudson and others have shown that discrimination is most costly in the presence of high SES [18], a finding which has been replicated for Black youth [49]. Of course, the solution is not to reduce the aspirations for Blacks but to eliminate discrimination towards them. Another solution is to equalize the cost of upward social mobility for minorities, so groups similarly gain from their upward social mobility [21,22].

While structural racism has a role in these diminished returns [74,75,76], other mechanisms may also have a role. Differential opportunities of Blacks and Whites in the labor market, purchasing power, extra-costs of upward social mobility, and residential segregation have a role in shaping disparities in gain from SES [11,12]. Some research has also shown that obesity has weaker effects on intention to reduce weight in Blacks than Whites which means Blacks with obesity are less ready to take action to control weight [77,78]. Therefore, additional support is required for reducing burden of obesity in the Black communities.

Given existing neighborhood/racial segregation [79], the same SES is associated with differentiating purchasing power by race [80]. Although we were unable to control for neighborhood segregation, the role of higher level factors such as density of fast food restaurants, food deserts, and availability of fresh food products on health of Whites and Blacks are well known [81,82,83]. Future research may explore multi-level factors that cause diminished return of SES on healthy diet and exercise as well.

The results of this study advocates for conceptualization of early interventions on social determinants of health that can reduce racial disparities in childhood obesity. Thus, to reduce disparities in adulthood, there is a need to have a life-course approach, and address barriers in the life of Blacks that increase risk of childhood obesity and then cardiovascular problems later in life. Late interventions may not be ideal to eliminate racial disparities, as such disparities shape early in life.

This finding on the protective effect of income to needs ratio against odds of childhood obesity for White children is in line with previous studies on protective effects of high SES against obesity [71,84,85]. Low SES has a wide range of negative health consequences and obesity is only one example [84,85,86,87,88,89]. Protective effects of SES extend to a wide range of physical and mental health outcomes [1,2,3].

Diminished return of SES is not specific to childhood [11,12]. A study showed education better changes drinking habits of Whites than Black older adults [28]. In the Health and Retirement Study (HRS), income had a protective effect against sustained high BMI among White and Black women, but not for White and Black men. In the same study, education had a protective effect against high BMI, physical inactivity, and insomnia for White men, White women, and Black women but not Black men [17]. Among adults, education [46], employment [90], neighborhood quality [69], and social contacts [91] generate smaller gain in life expectancy for Blacks than for Whites. All of these findings are in concert and support the Minorities’ Diminished Return theory, meaning the systematically smaller health gain due to SES for Blacks than Whites [17,44,46,92]. 

We are not arguing that Blacks and other disadvantaged racial/ethnic groups would not benefit from high SES and would in fact be harmed by it. Such argument could lead to disadvantageous interventions/policies targeted to these groups. Instead, we argue that the solution to disparities are going beyond equalizing access and enhancing minorities’ ability to turn their resources to tangible outcomes. Some example policies would be skill building that increase Blacks’ ability to compete with Whites to secure high paying jobs, and to invest on quality of education and transportation in inner cities that again can help Blacks with education secure more prestigious jobs. 

These results may have some implications for policy, practice, and research. Policy makers should be aware of diminished health return of SES for racial and ethnic minorities. These diminished returns are systemically neglected and cannot be solved simply by increasing access of the populations to resources. There is a need for policies and programs that help Blacks and other racial minorities to most effectively use their economic resources. There may be a need for programs that help Black families navigate the society and market and enhance their purchasing power. There may be a need to enhance walkability and availability of food in urban communities. Enhancing availability of healthy foods in highly segregated residential areas may be one of the specific solutions to reduce diminished return of SES in preventing childhood obesity for Blacks.

## 5. Limitations

Our study had some limitations. First, the study was cross-sectional in design, thus it is not possible to draw causative conclusions. However, it is unlikely that childhood obesity result in poor SES of family. However, there is a need for replication of these findings using a longitudinal design. Second, the SES indicators studied were not a comprehensive list, and other factors such as family structure, household size, employment, and wealth were not considered. Third, all the study measures were individual level, and there is a need for future research on contextual factors that these families are embedded in. Fourth, we focused on Blacks and Whites only, and did not include other potential moderators such as ethnicity, region, and neighborhood. There is a need for future research to replicate these findings across other marginalized groups such as Indian Americans, Hispanics, and immigrants. Fifth, this study used parent-reported height and weight to calculate child over-weight status. Sixth, age was used as a continuous variable, however, the relationships assessed here may differ for toddlers and teenagers. Further analysis of age is need. Seventh, we categorized our variables which necessarily reduces the information. This decision was made because income to needs ratio was only available in the current form. Information regarding categorical obesity may have more applied implications than data on BMI. Eight, the correlations were not very strong and variables did not explain a large amount of the variance of the dependent variable. Finally, we did not study cultural or behavioral mechanisms such as eating habits, hunger, breast feeding, or fast food. As the data were more than a decade old, there is a need to replicate these finding using current data. Despite these limitations, this study was one of the first ones to use a national sample of children to explore Black–White variation in the association between SES and obesity. 

## 6. Conclusions

Racial differences exist in the protective effect of income on childhood obesity. Due to the existing racism, Blacks consistently gain less from the very same SES resources. Reduction of residential segregation and enhancing availability of healthy foods in Black communities are some of the specific solutions to reduce Blacks’ diminished return of SES in preventing childhood obesity. Future research should study multi-level barriers that hinder Blacks from protective effects of SES resources against childhood obesity. To eliminate or at least reduce minorities’ diminished returns, we need more than equalizing access across of racial groups to SES. 

## Figures and Tables

**Table 1:** Descriptive statistics in the pooled sample and by race.

Characteristics	All (*n* = 76,705)	Whites (*n* = 67,610)	Blacks (*n* = 9095)
	*n*	%	*n*
	% (95% CI)	% (95% CI)	% (95% CI)
Child Race			
White	82.03 (81.47–82.58)	-	-
Black	17.97 (17.42–18.53)	-	-
Child Gender			
Male	51.06 (50.44–51.69)	51.42 (50.76–52.07)	49.46 (47.70–51.23)
Female	48.94 (48.31–49.56)	48.58 (47.93–49.24)	50.54 (48.77–52.30)
Parental Education *			
Less than high school graduate	4.07 (3.76–4.39)	3.38 (3.09–3.69)	7.21 (6.24–8.33)
High school graduate	25.39 (24.82–25.97)	23.31 (22.73–23.90)	34.87 (33.15–36.63)
More than high school	70.55 (69.94–71.15)	73.31 (72.69–73.93)	57.92 (56.12–59.69)
Child Obesity *			
No	75.86 (75.31–76.40)	78.63 (78.08–79.16)	63.23 (61.49–64.93)
Yes	24.14 (23.60–24.69)	21.37 (20.84–21.92)	36.77 (35.07–38.51)
	Mean (CI)	Mean (CI)	Mean (CI)
Child Age (Year)	9.65 (9.59–9.70)	9.63 (9.57–9.69)	9.73 (9.57–9.89)
Parental Education (1–3) *	2.64 (2.63–2.65)	2.68 (2.67–2.68)	2.49 (2.47–2.52)
Family Income to Needs Ratio *	5.41 (5.38–5.45)	5.74 (5.70–5.77)	3.91 (3.82–4.00)

Source: National Survey of Children’s Health (NSCH), 2003–2004; All numbers are weighted; * *p* < 0.05.

**Table 2:** Correlation matrix in the pooled sample and by race.

Characteristics	1	2	3	4	5	6
**All (*n* = 76,705)**						
1 Child Race (Blacks)	1.00					
2 Chile Gender (Females)	0.01	1.00				
3 Child Age (Year)	−0.01	0.00	1.00			
4 Parental Education (1–3)	−0.10	0.00	0.00	1.00		
5 Family Income to Needs Ratio	−0.21 *	0.00	0.07	0.42 *	1.00	
6 Childhood Obesity	0.12 *	−0.07	−0.26 *	−0.09	−0.13 *	1.00
**Whites (*n* = 67,610)**						
2 Chile Gender (Females)	-	1.00				
3 Child Age (Year)	-	0.00	1.00			
4 Parental Education (1–3)	-	0.01	0.01	1.00		
5 Family Income to Needs Ratio	-	0.00	0.08	0.40 *	1.00	
6 Childhood Obesity	-	−0.08	−0.26 *	−0.09	−0.11 *	1.00
**Blacks (*n* = 9095)**						
2 Chile Gender (Females)	-	1.00				
3 Child Age (Year)	-	0.00	1.00			
4 Parental Education (1–3)	-	−0.02	−0.02	1.00		
5 Family Income to Needs Ratio	-	−0.03	0.05	0.46 *	1.00	
6 Childhood Obesity	-	−0.05	−0.31 *	−0.06	−0.07	1.00

Source: National Survey of Children’s Health (NSCH), 2003–2004; All numbers are weighted; * *p* < 0.05.

**Table 3:** Summary of linear regression models in the pooled sample and by race.

Characteristics	Pooled Sample (*n* = 76,705)		Whites (*n* = 67,610)	Blacks (*n* = 9095)
	*Model 1*	*Model 2*	*Model 3*	*Model 4*
	*Main Effects*	*Main Effects + Interactions*	*Main Effects*	*Main Effects*
	OR (95% CI)	OR (95% CI)	OR (95% CI)	OR (95% CI)
Child Race (Blacks)	2.00 *** (1.83–2.19)	1.57 *** (1.25–1.98)		
Child Gender (Females)	0.70 *** (0.65–0.75)	0.70 *** (0.66–0.75)	0.67 *** (0.60–0.72)	0.82 * (0.70–0.96)
Child Age (Year)	0.86 *** (0.85–0.87)	0.86 *** (0.85–0.87)	0.86 *** (0.86–0.87)	0.85 *** (0.83–0.87)
Parental Education				
Less than high school graduate	Ref	Ref	Ref	Ref
High school graduate	0.96 (0.78–1.18)	0.90 (0.71–1.14)	0.90 (0.71–1.13)	1.09 (0.73–1.61)
More than high school	0.68 *** (0.55–0.83)	0.64 *** (0.51–0.81)	0.64 *** (0.51–0.81)	0.74 (0.50–1.10)
Family Income to Needs Ratio	0.94 *** (0.92–0.95)	0.92 *** (0.91–0.94)	0.92 *** (0.91–0.94)	0.98 (0.95–1.01)
Child Race * Parental Education				
Less than high school graduate	-	0.85 (0.54–1.34)	-	-
High school graduate	-	1.04 (0.84–1.28)	-	-
More than high school	-	1.00	-	-
Child Race * Family Income to Needs Ratio	-	1.06 ** (1.02–1.10)	-	-
Intercept	3.47 *** (2.75–4.36)	3.95 *** (3.06–5.10)	4.09 *** (3.16–5.29)	4.77 *** (2.93–7.77)

Source: National Survey of Children’s Health (NSCH), 2003–2004; Outcome: childhood obesity, Confidence Interval (CI); All numbers are weighted, * *p* < 0.05, ** *p* < 0.01, *** *p* < 0.001.
